# Arrestin-1 engineering facilitates complex stabilization with native rhodopsin

**DOI:** 10.1038/s41598-018-36881-4

**Published:** 2019-01-24

**Authors:** Raphael S. Haider, Florian Wilhelm, Aurélien Rizk, Eshita Mutt, Xavier Deupi, Christian Peterhans, Jonas Mühle, Philipp Berger, Gebhard F. X. Schertler, Jörg Standfuss, Martin K. Ostermaier

**Affiliations:** 1 InterAx Biotech AG, PARK InnovAARE, Villigen, 5234 Switzerland; 2 Laboratory of Biomolecular Research, Paul Scherrer Institute, Villigen, 5232 Switzerland; 3 Present Address: Institute of Molecular Cell Biology, Jena, 07745 Germany; 4 ETH Zurich, Zurich, 8093 Switzerland

## Abstract

Arrestin-1 desensitizes the activated and phosphorylated photoreceptor rhodopsin by forming transient rhodopsin−arrestin-1 complexes that eventually decay to opsin, retinal and arrestin-1. Via a multi-dimensional screening setup, we identified and combined arrestin-1 mutants that form lasting complexes with light-activated and phosphorylated rhodopsin in harsh conditions, such as high ionic salt concentration. Two quadruple mutants, D303A + T304A + E341A + F375A and R171A + T304A + E341A + F375A share similar heterologous expression and thermo-stability levels with wild type (WT) arrestin-1, but are able to stabilize complexes with rhodopsin with more than seven times higher half-maximal inhibitory concentration (IC_50_) values for NaCl compared to the WT arrestin-1 protein. These quadruple mutants are also characterized by higher binding affinities to phosphorylated rhodopsin, light-activated rhodopsin and phosphorylated opsin, as compared with WT arrestin-1. Furthermore, the assessed arrestin-1 mutants are still specifically associating with phosphorylated or light-activated receptor states only, while binding to the inactive ground state of the receptor is not significantly altered. Additionally, we propose a novel functionality for R171 in stabilizing the inactive arrestin-1 conformation as well as the rhodopsin–arrestin-1 complex. The achieved stabilization of the active rhodopsin–arrestin-1 complex might be of great interest for future structure determination, antibody development studies as well as drug-screening efforts targeting G protein-coupled receptors (GPCRs).

## Introduction

Arrestins are most prominent interaction partners for G protein-coupled receptors (GPCRs) and play an essential role as signal-terminating proteins. By binding to the core region of the receptor, arrestins sterically hinder the agonist-activated GPCR to induce further G protein-dependent signaling^1^. Upon ligand-binding of the GPCR and its subsequent conformational change, phosphorylation of cytoplasmic sites of the GPCR initiates the activation of arrestin^2^. Arrestins are then able to either engage the receptor at the intracellular core region, formed by the bundle of seven transmembrane helices^3^, or associate with the receptor via the phosphorylated GPCR C-terminus only^4,5^. Both complexes have been shown to allow arrestins to modulate receptor signaling as well as trafficking^6,7^. Due to the sheer number of GPCRs that depend on regulation by arrestins and their differences in structure and sequence, arrestin proteins show widely varying affinities towards particular receptors: The two arrestins of the visual system, arrestin-1 and arrestin-4, bind to either agonist-bound rhodopsin or color opsins with high affinities^8,9^. Contrarily, arrestin-2 and arrestin-3 form complexes with GPCRs of different interaction strengths depending on the recruiting GPCR, its conformation and the level of its phosphorylation^10,11^. Several phosphate-sensing regions have been identified in the arrestin protein to be of major importance for receptor recognition and the promotion of high affinity binding^12–14^.

Due to its abundance in natural sources and its low conformational flexibility, rhodopsin was the first GPCR to be structurally analyzed and still serves as a model system for the structural characterization of GPCRs^15^. Several spectrophotometric intermediates have been trapped and described by various techniques in order to explain the activation path from retinal photo-isomerization to formation of the active G protein-binding conformation^16^. Roughly one third of all solved GPCR structures show different activational and conformational states of rhodopsin. The malfunctioning of the visual system, which features rhodopsin, G protein-coupled receptor kinase 1 (GRK1) and arrestin-1, may lead to retina degeneration by retinitis pigmentosa^17^ or to night blindness^15,18–22^. The system at hand therefore also serves as a relevant pharmacological target^23^.

Key regulation sites and conformational dynamics of arrestin activation and recruitment have been most intensively studied for the visual system, composed of arrestin-1 and rhodopsin^24–28^. Previously, we have presented a comprehensive functional map of single residue contributions in arrestin-1 to the binding of light-activated and phosphorylated rhodopsin^14^. Plotted on crystal structures of the arrestin-1 ground^29^ and pre-activated states^30,31^, our data allowed a detailed functional analysis at single amino acid resolution. We revealed the relative impact of phosphorylation-sensing residues, of residues anchoring the arrestin C-terminal tail onto the N-domain and of key residues within the polar core^29,32^. Further, the relative impact of residues of various loops that have been proposed to sense the “activation state”^28,33–35^ of the corresponding receptors have been revealed. A recent study performed by Sente *et al*. combined this functional map of arresin-1 with extensive computational analysis and confirmed multiple residues as crucial regulators for phosphorylation-sensing, arrestin activation and receptor-binding^36^. Mutational studies on the arrestin gene and exploration of various modifications and their effect on affinity of GPCR-binding have been of interest as seen in studies for instance, conducted by Gurevich *et al*.^37–39^. Studies of this kind also suggest the possibility of functional modification of arrestin-1 for compensational gene therapy in case of hyperactive or phosphorylation-deficient GPCRs^40,41^.

In this work, we selected 57 mutants from the previously accomplished library of 403 single-point alanine/glycine arrestin-1 mutants^14^ and subjected them to a multi-dimensional screening and combination strategy. We generated 53 double, 38 triple and 15 quadruple mutants by scanning mutagenesis^42^ and assayed them in the high-throughput manner for binding to light-activated and phosphorylated rhodopsin as previously described^14^. Thus, we derived half-maximal inhibitory concentration (IC_50_) values of sodium chloride for the formation of rhodopsin−arrestin-1 mutant complexes. Additionally, we determined the expression level of functional and rhodopsin-binding arrestin-1 proteins for each mutant in bacterial cells, in the following called “functional expression level”. We introduced an in-gel fluorescence-based thermo-stability assay to measure melting temperatures (T_M_) of selected arrestin-1 mutants. We probed selected arrestin-1 mutants for binding to five different rhodopsin states to reveal the functional impact of mutations that were iteratively combined. Modeling and molecular dynamics simulations allowed us to derive structure–function relationships of selected mutants and in particular, to shed light on the dual functionality of position R171.

## Results

### Arrestin protein engineering

In an attempt to characterize critical amino acids influencing rhodopsin–arrestin interactions, we previously conducted comprehensive scanning mutagenesis on the arrestin-1 protein, in which every amino acid of the protein was substituted by alanine or, in the case of a native alanine residue, by glycine^13,14,42^. Subsequently, the influence of every single residue on rhodopsin–arrestin binding was measured and quantitatively compared as to shed light on arrestin-1 activation and complex formation with rhodopsin^13,14,43^. In this study, we utilize this previously established functional map of single residue contributions in arrestin-1 for rhodopsin–arrestin-1 complex formation. We present a multidimensional strategy to create combined arrestin-1 mutants designed for higher receptor affinity. These mutants could be used in different applications, such as crystallography^3^, gene therapy^44^, antibody-development or drug screening. Advances in creating a stable, high-affinity rhodopsin–arrestin complex have already been made by strategic mutagenesis^45^, utilizing the accumulated knowledge of arrestin mutations gathered over the course of more than 20 years^44^. In this novel protein engineering approach, which is potentially applicable to any mutable binding partner of a GPCR, we demonstrated a dramatic increase in speed to create proteins with desired functionalities compared to traditional methods. Here, the binding strength of a particular arrestin-1 mutant, C-terminally fused to mCherry, was determined by measuring the fluorescence intensity after a pull-down with membranes containing phosphorylated as well as light-activated rhodopsin.

**Figure 1:**
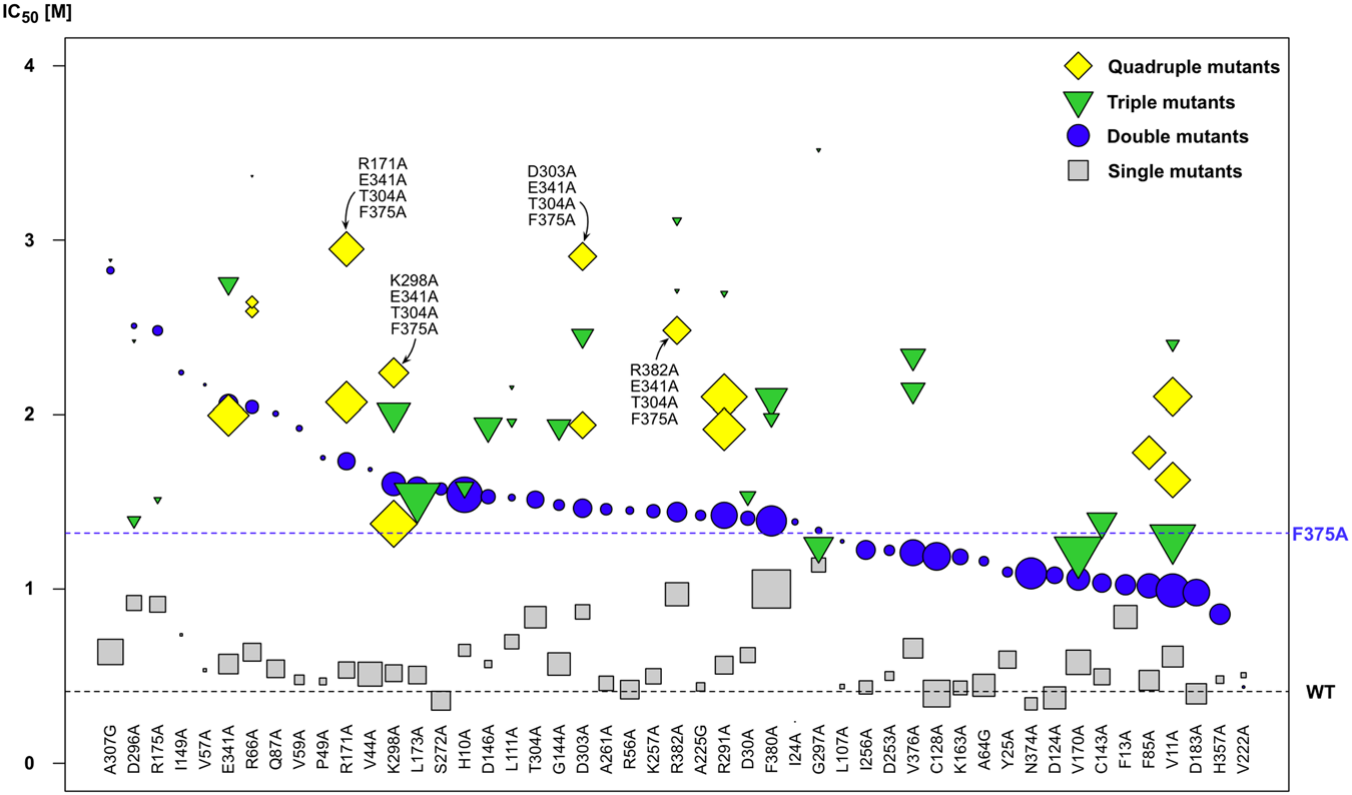
Half-maximal inhibitory concentration (IC_50_) values of sodium chloride for formation of complexes composed of mutant arrestin-1 and both phosphorylated and light-activated rhodopsin. Double mutants (blue circles) are sorted by decreasing IC_50_ value (from left to right) and are composed of F375A + X, where the single mutant X is shown underneath and named on the x-axis. Triple mutants are composed of either F375A + A307G + X or F375A + T304A + X, while quadruple mutants are combinations of F375A + T304A + E341A + X or F375A + T304A + F380A + X. The size of the shape encodes the functional expression level of the respective mutant and is scaled in relation to the WT arrestin-1 functional expression level (legend on the top right). IC_50_ values, functional expression level of single^13,14^ and combined mutants are listed in SI Table 1.

The library of 403 arrestin-1 mutants was screened for candidates showing the highest IC_50_ values for rhodopsin–arrestin-1 complex stability under the pressure of ionic strength (NaCl), in order to find mutant combinations that would increase binding. We accordingly selected 23 mutants for further combination attempts due to their WT-like expression patterns and high IC_50_ values, ranging from 1.14 M NaCl for G297A to 0.56 M for R291A^13,14^. These mutations were combined with the most complex-stabilizing mutation F375A (IC_50_ 1.32 ± 0.31 M) in order to create an array of arrestin-1 double mutants. F375A also showed an acceptably high level of functional protein expression (107 ± 26% when compared to the WT) and a thermo-stability value comparable to the WT arrestin protein (T_M_ 63.3 ± 1.0 °C). Our observations for WT-like stability and increased affinity of the F375A mutant are in good agreement with previous examinations^39^. Beside those 23 double mutants, another 15 mutants with significantly higher IC_50_ values than the WT, as well as ten mutants with WT-like IC_50_ values and five mutants, with significantly lower IC_50_ values or low functional expression, were selected as controls and also combined with F375A. Thus, a total of 53 arrestin-1 double mutants were created and subsequently tested for their affinity towards rhodopsin at various concentrations of sodium chloride (Fig. 1).

The design strategy for high-affinity arrestin-1 mutants was influenced by plans for future biotechnological applications; for example, their usage in surface plasmon resonance studies, antibody development, structural biology or cellular assays, as recently reviewed in^46^. Hence, mutants were selected not only for their binding capabilities but had to show a high functional expression level and WT-like thermo-stability. After the characterization of 53 double mutants, the goal was to introduce further mutations to identify triple and quadruple mutants, showing high rhodopsin affinity as well as WT-like protein expression and stability.

Functional expression levels were measured alongside IC_50_ values, as each batch of assessed mutants was expressed, measured and compared to a WT arrestin-1 control. Numerical values for expression were directly derived from the strength of fluorescence signal obtained from each individual measurement and compared to the WT arrestin-1 control, which was measured side by side^13,14^. In this study, we found that low functional expression levels are introduced by single point mutations and cannot be easily rescued by adding further mutations that cause the protein to express WT-like levels. For example, combinational mutants containing the A307G mutation showed exceptionally high IC_50_ values (Fig. 1), but we excluded these mutants and others from further combinations because we identified arising problems for downstream methodologies that may require higher amounts of protein, such as protein crystallization. Interestingly, seven out of eleven double mutants that bore low expression levels, had IC_50_ values significantly higher than the F375A single mutant. We presume that such mutations increase the flexibility of the arrestin molecule and thereby facilitate binding to rhodopsin, but make the protein prone to degradation due to low conformational stability or disturbance of the protein fold. Other mutations, such as F380A, showed a significantly higher expression level compared to WT arrestin-1 and were subsequently introduced into further mutant combinations, in the attempt to balance the functional expression and rhodopsin–arrestin mutant binding levels.

**Figure 2:**
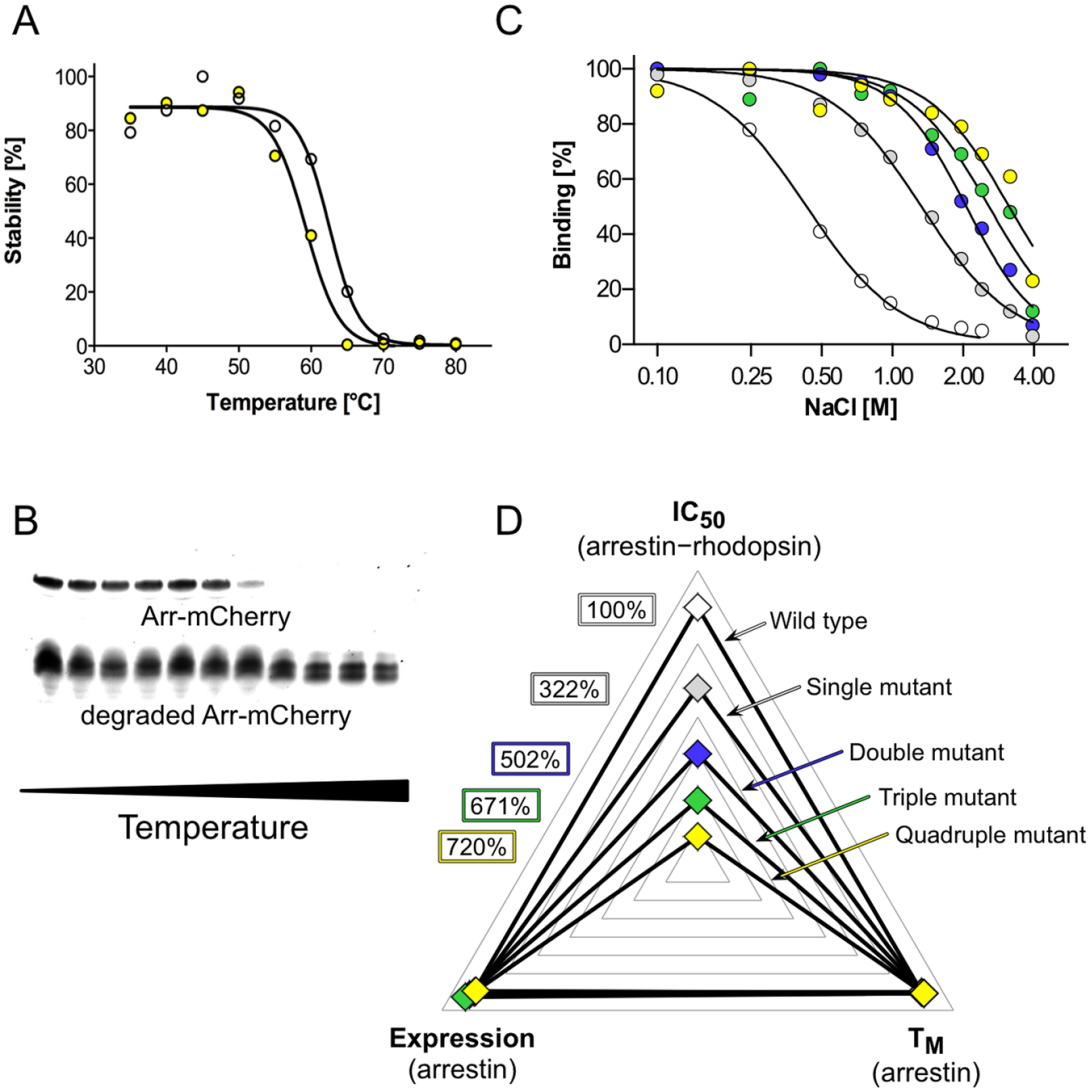
Arrestin-1 mutant combination strategy and multi-dimensional screening. (**A**) Arrestin–mCherry protein T_M_ detected by in-gel fluorescence for WT arrestin-1–mCherry (white circles) and quadruple arrestin-1–mCherry mutant R171A + E341A + T304A + F375A (yellow circles). (**B**) SDS in-gel fluorescence signal of an arrestin-1–mCherry quadruple mutant (R171A + E341A + T304A + F375A) decreases upon temperature-induced denaturation. In contrast, signal from degraded/aggregated arrestin-mCherry stays constant during the procedure. (**C**) Arrestin-1 mutants were combined in order to successively increase IC_50_ values. WT arrestin-1 (white circles), single mutant F375A (grey circles), double mutant E341A + F375A (blue circles), triple mutant E341A + T304A + F375A (green circles), and quadruple mutant D303A + E341A + T304A + F375A (yellow circles) are shown. (**D**) We selected and combined mutants with increased IC_50_ values, but with T_M_ values and functional expression levels close to WT values. Color codes and mutants are the same as in (**C**). IC_50_ and T_M_ values as well as functional expression levels are available in SI Table 1 and are normalized to WT arrestin-1.

We established an in-gel fluorescence thermo-shift assay in order to rapidly identify mutations characterized by undesirable destabilizing effects on the arrestin-1 protein in terms of thermo-stability without the need to purify each individual mutant protein (Fig. 2A,B). The cleared bacterial cell lysate containing a particular arrestin-mCherry mutant was exposed to a heat gradient and aggregated and degraded protein was subsequently removed by centrifugation. Remaining protein in solution was separated by sodium dodecyl sulfate polyacrylamide gel electrophoresis (SDS-PAGE). Quantification of full-length arrestin-mCherry WT or mutants by in-gel fluorescence and Boltzmann sigmoidal fitting of obtained data allowed us to derive T_M_ values. With this method we determined the T_M_ of 25 single mutants as well as the WT arrestin-mCherry construct. Only one mutant, namely F377A, showed with 66.0 ± 1.3 °C a slight tendency for a higher T_M_ than the WT with 64.2 ± 0.6 °C. Five mutants showed T_M_ values comparable to the WT, whereas 19 mutants exhibited significantly lower T_M_ values than the WT protein ranging from 62.4 ± 1.0 °C for L173A down to 52.5 ± 0.7 °C for R175A (SI Table 1). Similar observations for C-tail mutants of arrestin-1 have been previously reported utilizing an approach where protein has been exposed to elevated temperature, separated from aggregated protein by centrifugation and tested for binding to rhodopsin^39^. For most cases in this study, the screened double mutants showed T_M_ values that were slightly below the averaged T_M_ values of corresponding single mutants. This effect was often observed in combinational mutants and seems to accumulate with higher number of mutations. Thus, it was necessary to monitor changes in functional expression, thermo-stability and in affinity towards phosphorylated as well as light-activated rhodopsin.

**Table 1:** Arrestin-mCherry fusion constructs.

Mutants	Binding capability^a^	Functional expression level relative to WT	Activity level relative to WT
WT	**100%**	**100%** (s.d.: N/A, n: 74)	**100%** (s.d.: 16, n: 5)
D303A, T304A, E341A, F375A	**710%**	**101%** (s.d.: 20, n: 4)	**44%** (s.d.: 16, n: 5)
R171A, T304A, E341A, F375A	**720%**	**127%** (s.d.: 12, n: 4)	**88%** (s.d.: 27, n: 8)
T304A, E341A, F375A	**671%**	**68%** (s.d.: 16, n: 3)	**149%** (s.d.: 55, n: 4)
T304A, F375A	**502%**	**86%** (s.d.: 8, n: 3)	**106%** (s.d.: 31, n: 4)
F375A	**322%**	**106%** (s.d.: 26, n: 17)	**102%** (s.d.: 20, n: 4)

^a^Increase of IC_50_ value, indicating complex stability under the pressure of ionic strength, compared to WT.

Thermo-stability as well as functional expression levels of each individual mutant in mind, we added another series of mutations on top of double mutant A307G + F375A, leading the screen with the highest IC_50_ value of 2.83 M, and T304A + F375A, which was with an IC_50_ value of 1.51 M under the twelve best-binding mutants. Out of 14 triple mutants containing A307G and F375A mutations, ten bound in low or not detectable amounts, when examined for rhodopsin affinity. Out of 25 triple mutants based on T304A + F375A, only four showed this kind of low functional expression level. Thus, we excluded constructs containing mutation A307G for further mutagenesis, although these mutants exhibited the highest IC_50_ values observed so far. For the creation of quadruple mutants we chose triple mutants E341A + T304A + F375A and F380A + T304A + F375A. The former combinational mutant was chosen because of its exceptionally high IC_50_ value of 2.75 M, whereas the latter was used for further mutant combination due to its high level of functional expression. Both triple mutants showed acceptable T_M_ values of around 60 °C. Finally, we subjected both triple mutants to further mutagenesis and found quadruple mutants D303A + E341A + T304A + F375A and R171A + E341A + T304A + F375A, which exhibit IC_50_ values amounting to 720% and 710% of the WT value, respectively (Fig. 1; Fig. 2 and SI Table 1). Conclusively, we were able to increase complex stability to more than seven times under the pressure of ionic strength, but preserve WT-like functional expression levels (Figs. 1 and 2C,D).

### Structural location of selected arrestin-1 mutants

**Figure 3:**
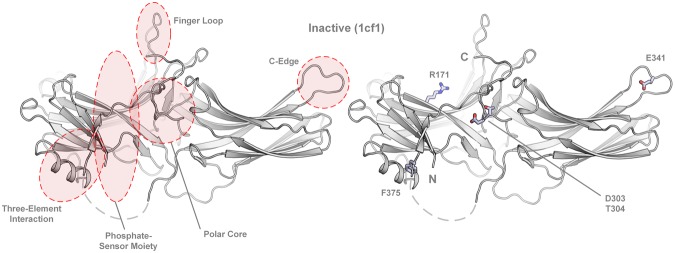
Location of crucial domains as well as the mutated amino acids, as indicated in the inactive (1cf1, α conformer)^29^ arrestin-1 structure. The binding-enhancing alanine substitutions at positions R171, D303, T304, E341 and F375 are shown as sticks.

The structural investigation of the five most complex-stabilizing mutations (F375A, D303A, T304A, E341A and R171A), confirms the importance of several specific sites in the arrestin protein for the binding of phosphorylated as well as light-activated rhodopsin. The most important structural domains along with the positions of the mutated amino acids in the inactive conformation of arrestin-1 are shown in Fig. 3.

From a structural aspect, F375 is located in the C-terminal tail and shields the hydrophobic three-element interaction site composed of α-helix I, β-sheet I and distal C-terminal tail of arrestin-1. The disruption of interactions at this site increases the flexibility of the C-terminal tail and will ultimately lead to the destabilization of the polar core. This process (presumably facilitated by phosphate moieties of the GPCR C-terminus^47^) lowers the energy barrier between the basal and active arrestin conformation, as demonstrated by crystallization of an arrestin-1 splice variant in either basal^48^ or pre-activated form^30^ as well as atomic-level simulations^49^. Thus, the F375A mutation reduces the selectiveness for phosphorylated sites^39^ of the GPCR and makes arrestin prone to be activated, while maintaining WT-like thermo-stability and functional expression level of the protein^45^. Mutation T304A is located in the polar core next to D303, a crucial amino acid that stabilizes the basal activation state of arrestin-1 by forming a salt bridge with R175. Interestingly, mutations with most severe effects on thermo-stability, resulting in a T_M_ decrease of 5 to 11 °C, were located within the polar core (R175A, D303A) or structurally close to it (D296A, G297A and A307G). These findings again confirm that mutations in the polar core strongly influence protein stability and disposition towards GPCR-binding conformations.

E341 is located in the C-edge of arrestin-1, which undergoes structural rearrangements upon contact of arrestin-1 with the phosphorylated C-terminal tail of rhodopsin^50^. The main function of the C-edge region was a subject of speculation until it was identified to serve as a membrane anchor supporting the GPCR-arrestin complex formation upon arrestin-1 pre-activation^26,50^. While mutation of the mostly hydrophobic residues at the C-edge results in weaker binding of arrestin to the receptor, the alanine substitution of E341 leads to elevated binding, presumably due to the abolishment of attractive or repulsive forces with polar membrane head groups^14,50^. It remains to be analyzed whether the E341A mutation has any influence on the oligomerization state of arrestin quadruple mutants^51–53^.

Residue R171 might play crucial roles in the C-tail exchange mechanism. It has already been shown that the R171Q mutation reduces the affinity of arrestin-1 towards phosphorylated forms of rhodopsin^54^ and was hypothesized to interact with phosphate moieties of the C-terminus^55^. A recent study, interpreting a high-resolution crystal structure of the rhodopsin–arrestin-1 complex (crystalized as a single fusion protein), showed that R171 is indeed directly interacting with the phosphorylated T336, located in the rhodopsin C-terminal tail^26^.

### Arrestin-1 mutants show improved binding affinities towards distinct (rhod-)opsin states

**Figure 4:**
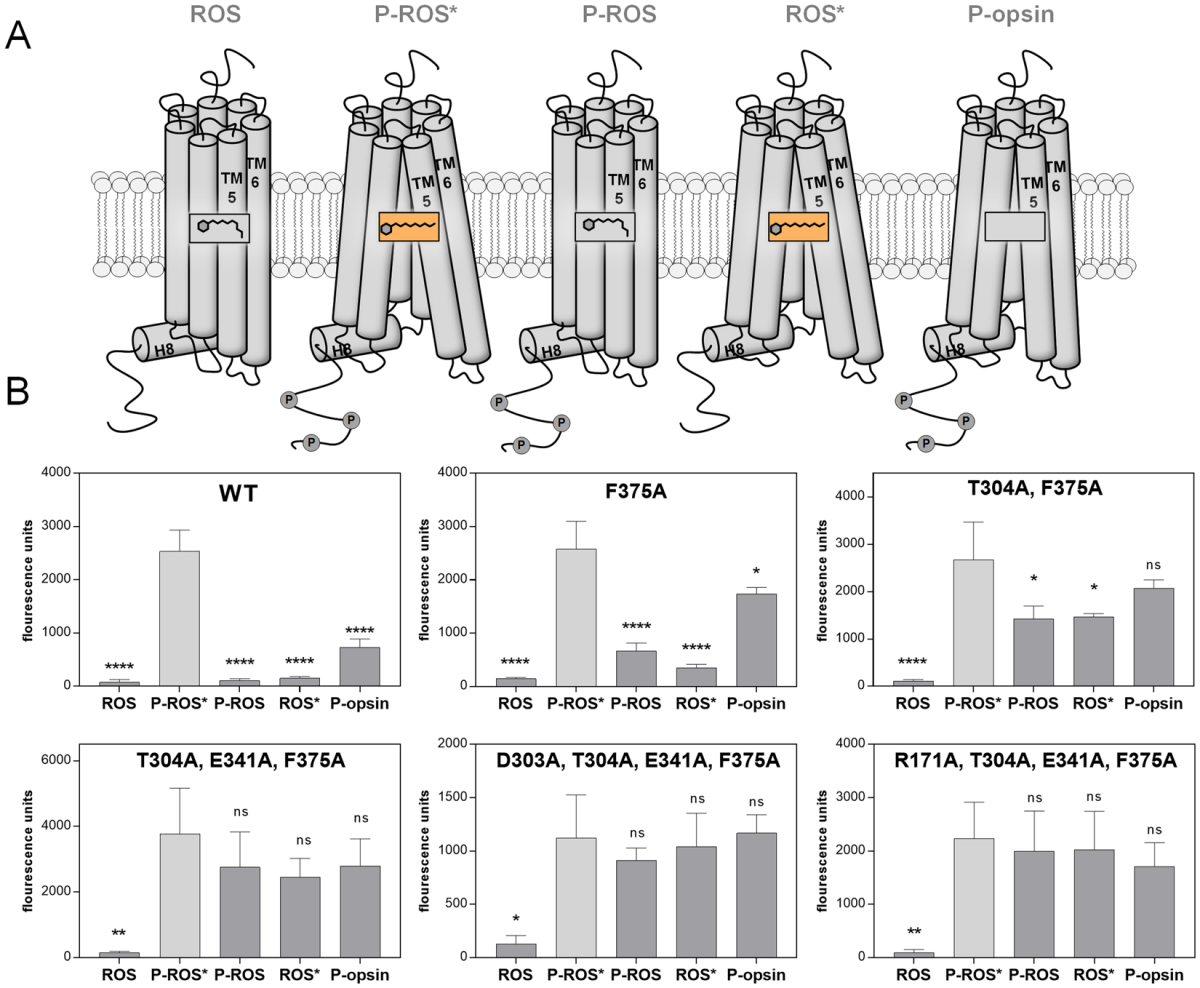
(**A**) Schematic depiction of the (rhod-)opsin activation and phosphorylation states contained within ROS membranes. Light-activated states of the receptor are indicated via a box containing the structure of the chromophore, retinal. (**B**) WT, single, double, triple and two quadruple mutant arrestin-mCherry fusion proteins were expressed in HEK293 cells and purified. Each protein was used in a direct binding assay with membranes containing the depicted activation and phosphorylation states of the (rhod-)opsin receptor. Fluorescence units indicate the level of bound arrestin-1–mCherry fusion protein to the specific receptor state after membrane pull down and two washing steps. Membranes containing P-ROS, ROS and P-opsin were kept in the dark throughout the experiment, whereas P-ROS* and ROS* were illuminated for 2.5 min using light, filtered with a 495 nm long-pass filter before pull down. The experiments were carried out in triplicates and normalized to the amount of purified arrestin-mCherry protein (1.5 µg). The statistical significance of measurements is indicated in comparison with P-ROS* binding and determined by one-way ANOVA: *p < 0.05; **p < 0.01; ***p < 0.001; ****p < 0.0001.

In order to further characterize the engineered arrestin-1 mutants we carried out another series of experiments utilizing an adjusted version of the direct arrestin binding assay^13,14^. Yet, instead of directly using bacterial cell lysate to perform the pull-down reactions, we used HEK293 cell-expressed, one-step purified arrestin-1–mCherry protein for reactions with controlled protein amounts. The mutants were measured against five distinct (rhod-)opsin activation/phosphorylation states contained in rod outer segment (ROS) membranes. Binding capabilities of the purified proteins to the light-activated (ROS*) state of the receptor were compared to values for the dark state (ROS), the light-activated and phosphorylated state (P-ROS*), the phosphorylated dark state (P-ROS) and the phosphorylated state of the apo-protein of the receptor, opsin, without any retinal bound (P-opsin). A schematic depiction of the putative receptor states, utilized in the assay, is shown in Fig. 4A. This characterization was performed on five mutants of interest and the mCherry-labeled WT arrestin-1. Detailed information on the selected mutants can be obtained from Table 1. Additionally, we produced saturation curves of the mutants F375A, D303 A + E341A + T304A + F375A and R171A + E341A + T304A + F375A for binding to P-ROS*, shown in SI Fig. 1.

The measurements indicate that all of the assessed arrestin-1 mutants as well as the WT arrestin-1 protein are characterized by a strong binding capability towards P-ROS* (Fig. 4B). On the other hand, the inactive and unphosphorylated receptor state (ROS) did not bind any of the applied arrestin-1 proteins at a high rate and always shows a significant difference in comparison to P-ROS*.

Changes of the binding properties due to arrestin-1 engineering can be seen for the P-ROS, ROS* and P-opsin states of the receptor (Fig. 4B**)**. For these receptor states, the binding of WT arrestin-1 only occurs at a very basal level, similar to the inactive and unphosphorylated state of the receptor. Notably, our data suggest that the arrestin-1 WT protein is characterized by a higher affinity towards the P-opsin state than towards the ROS, P-ROS and ROS* states. The arrestin-1 F375A mutant shows a very similar binding pattern to the assessed receptor states. Whereas the binding to P-ROS, ROS* and P-opsin is elevated in comparison to arrestin-1 WT, the values obtained for the binding to these states are still significantly lower than the ones obtained for the binding to P-ROS*. Upon introduction of a second, binding enhancing mutation, the T304A + F375A double mutant binds to P-ROS and ROS* still at a significantly lower level than to P-ROS* but shows a stronger binding towards P-opsin. The difference between the binding of the double mutant to P-opsin and the reference, P-ROS*, is not statistically significant. Furthermore, the addition of a third or fourth, in NaCl binding enhancing mutation increases the binding of arrestin-1 to P-ROS and ROS* to P-ROS*-like levels. Binding of the three tested mutants F375A, D303A + E341A + T304A + F375A and R171A + E341A + T304A + F375A to P-ROS* appeared to have similar modes and was saturable at similar maxima (SI Fig. 1). In detail, mutant F375A shows a slightly higher potency than the quadruple mutants (SI Fig. 1), reflecting the higher activity level of F375A compared to the quadruple mutants (Fig. 4B). Triple mutant E341A + T304A + F375A shows the highest activity of all five measured mutants compared to WT. In summary, increasing levels of engineering reduces the sensitivity of arrestin-1 for binding to typically non-preferred receptor states, except for ROS.

## Discussion

**Figure 5:**
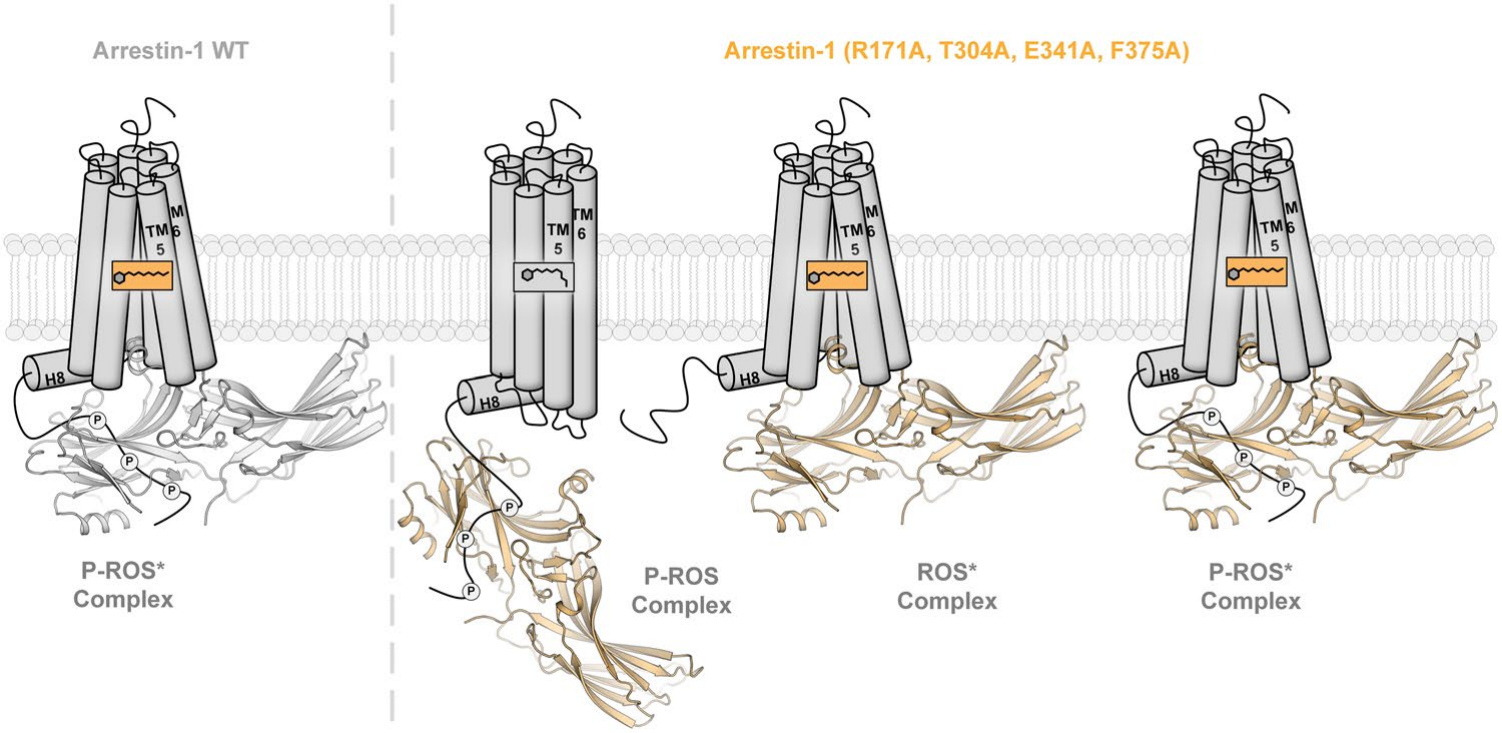
Proposed complex formation of the promiscuous arrestin-1 quadruple mutant with P-ROS and ROS* in comparison to the arrestin-1 WT bound P-ROS*. WT arrestin-1 has been shown to need both, electrostatic interactions via the GPCR C-terminal tail, as well as the conformational docking with the GPCR cytoplasmic cavity to form a stable interaction with rhodopsin. The shown data suggest that the arrestin-1 (R171A, T304A, E341A, F375A) quadruple mutant is additionally able to utilize both interactions individually to form a stable rhodopsin–arrestin-1 complex.

Combined, the obtained results suggest that the described arrestin-1 mutants bind to the (rhod-)opsin activation states at least as strongly as the WT or at elevated levels. Especially the enhanced binding to P-ROS and ROS* shows that the mutants are losing the capability to discriminate between those receptor states and P-ROS*. With a higher number of implemented mutations, this effect is amplified. On the other hand, the mutants still retain selectivity for activated and partially activated rhodopsin states, as the binding of the inactive ROS state shows no major elevation in comparison to WT arrestin-1. The binding capabilities of WT arrestin-1, demonstrated in this study, show that the protein needs both the electrostatic interaction with phosphorylated C-terminal GPCR residues and an active receptor conformation to achieve high complex stability. It has been shown that arrestins are able to associate with GPCRs in a tight complex with the cytosolic receptor cavity^3^ or with the C-terminus in a complex, mostly mediated by electrostatic interactions between phosphorylated C-terminal residues and the arrestin N-domain^4^. By introducing up to four complex stabilizing alanine mutations, the discussed arrestin-1 mutants seem to deviate from the arrestin-1 WT binding pattern and show the ability to associate with P-ROS and ROS* by forming one of the aforementioned complex-types, neglecting either the need for conformational complementation or electrostatic interaction with the GPCR C-terminal tail. A schematic depiction of these different binding modes, utilized by arrestin to form complexes with various rhodopsin species, is shown in Fig. 5.

We further analyzed mutants D303A + E341A + T304A + F375A and R171A + E341A + T304A + F375A in comparison to mutant F375A in titration measurements with fixed amounts of P-ROS* to better understand the diminished activity level of the quadruple mutants (Fig. 4B). However, binding modes and saturable maxima appear to be similar (SI Fig. 1). Only the potency of F375A seems to be slightly higher compared to the other quadruple mutants. Triple mutant E341A + T304A + F375A shows an even higher activity level compared to the quadruple mutants, but forms less stable complexes with rhodopsin in high salt concentrations. An increasing NaCl concentration may primarily select for arrestin mutants that form lasting complexes with various states of rhodopsin and thus, altogether over a longer time period. In the light of recent findings made by Eichel *et al*. in 2018, it is also conceivable that the presented arrestin-1 mutants are catalytically activated^56^, accumulate at the membrane and are thus pulled down without the formation of a prolonged receptor–interaction. With this generated data, we cannot exclude differences in the binding kinetics between the different mutants for binding to P-ROS*. The employed pull-down assay exceeds the anticipated half-life time frame of the arrestin-1–P-ROS* complex. Hence, it cannot truly reflect k_on_ and k_off_ values (Bayburt *et al*., JBC 2011). It would be interesting to measure those values in a time-resolved binding assay, to potentially discover the differences between the quadruple and triple mutants engaging with and dismissing rhodopsin.

Enhanced binding to the P-ROS and ROS* states, while still being able to discriminate between them and the inactive ROS conformation, could greatly benefit numerous biotechnological applications. This distinct feature of the analyzed arrestin-1 mutants could enhance sensitivity of high-throughput approaches characterizing the arrestin recruitment capabilities of a multitude of possible receptor-activating compounds at stable parameters. Especially for the assessment of a multi-step process like GPCR activation at constant conditions, these mutant properties could enable signal readouts for compounds that show weak GPCR binding or only induce transient or weak arrestin recruitment to the receptor. The P-opsin state represents the receptor conformation after activation and dissociation of the light-sensitive retinal^57^. Due to the enhanced binding capabilities of the studied arrestin-1 mutants of this particular receptor activation state, the mutant arrestin proteins might also show a prolonged residence time at the GPCR and thus could help to visualize rather transient arrestin–receptor complex formations.

In addition to these technological applications of the established arrestin-1 mutants, further studies still have to address the question of their functionality. Especially with recent findings addressing the functional competence of different GPCR–arrestin complexes^6,7^, arrestin mutants that are able to stabilize more than one distinct GPCR conformation might cause changes in receptor trafficking, fate or signaling. Further assessment of these complex enhancing arrestin-1 mutants might also include their potential to stabilize complexes with other GPCRs than (rhod-)opsin, since their higher affinity to various (rhod-)opsin states would also suggest a higher promiscuity to a variety of different GPCR conformations, in general. Following this idea, it should also be possible to transfer the presented alanine substitutions to β-arrestins in order to study their effect on the stability of other GPCR–arrestin complexes.

### Structure-function relationship of the arrestin-1 R171A mutant

Arrestin mutants showing a lower sensitivity to GPCR phosphorylation or, in other words, a higher affinity to non-phosphorylated receptors have been termed “pre-activated”^55^. The use of arrestins especially designed to fit this characteristic has led to enormous advances, for example the first elucidation of a crystal structure showing the complex of arrestin-1 with rhodopsin^3^. For the succeeding crystal structure, the arrestin-1 triple-A, alias 3A, F375A + V376A + F377A mutant was used, which also carries the F375A mutation, essential to all combination mutants described in the present study. In order to further characterize the properties of arrestin mutants, interactions of various engineered versions of arrestin-1 with different activation states of rhodopsin have been shown in this study.

A recent study, carried out by Zhou *et al*. in^26^, elucidated the interface between the phosphorylated rhodopsin C-terminal tail and the arrestin-1 N-domain by analyzing an X-ray free-electron laser crystal structure of the rhodopsin–arrestin-1 complex^26^. Due to this accomplishment, nine positively charged amino acids, located in the arrestin-1 N-domain, have been shown to directly interact with phosphorylated or negatively charged amino acids of the C-terminal tail of rhodopsin. Previously we presented a functional map of arrestin-1 at single amino acid resolution^14^ and were now able to correlate these data with the crucial, positively charged arrestin-1 amino acids of the binding interface (Table 2).

**Table 2:** Arrestin-1 mutations at positions directly involved in the C-terminal GPCR–arrestin-1 binding interface.

Mutants (bovine arrestin-1)^26^	IC_50_ value determined by Ostermaier *et.al*.^14^	Effect on binding^a)^^14^
K14A	0.30	73%
K15A	0.33	81%
R18A	0.33	81%
R29A	0.24	59%
K110A	0.37	90%
K166A	0.38	93%
K167A	0.36	88%
R171A	0.54	**132%**
K300A	0.33	81%
WT	0.41	**100%**

^a^Change of IC_50_ value, indicating complex stability under the pressure of ionic strength, compared to WT.

**Figure 6:**
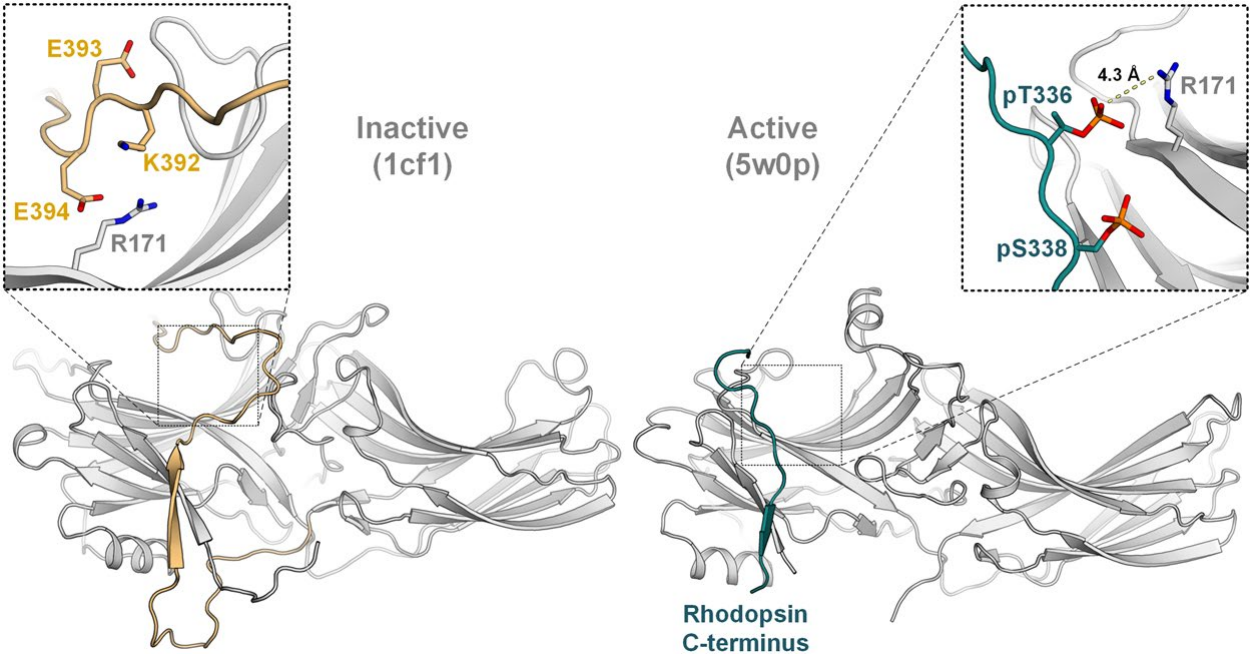
Comparison between possible electrostatic interactions of R171 in the inactive (1cf1, β confomer) and active (5w0p) arrestin-1 structures. On the left, the inactive conformation of arrestin-1 is shown^29^, featuring the modelled distal C-terminal tail in gold. The close-up view above indicates the proposed locations as well as orientations for charged, C-terminal amino acids in a 10 Å radius around R171. The rhodopsin C-terminal tail and the resolved phosphorylated residues are shown in teal, as elucidated in the crystal structure^26^.

As expected, alanine substitutions at all of the described positions lead to a decrease of complex stability. Only the mutation of R171, which was found to associate with the leading phosphorylated amino acid of the rhodopsin C-terminal tail, pT336^26^, results in an enhanced binding of 132% compared to the WT arrestin-1 protein^14^. This feature also led to the incorporation of R171A into one of the quadruple mutants, assessed in this study. In order to further uncover the structure–function relationship of R171 in the inactive arrestin conformation, a computational model was created to approximate the location of negatively charged residues of the distal arrestin-1 C-terminal tail. The model is based on the β conformer of the original arrestin-1 crystal structure^29^, while the sequence of the modeled C-terminal tail of bovine arrestin-1 was obtained from the Uniprot database (Uniprot:P08168). A depiction of possible charge–charge interactions for R171 in the rhodopsin-bound and the modelled, inactive arrestin-1 structure is shown in Fig. 6.

Since the disruption of a seemingly essential binding component of the rhodopsin–arrestin-1 complex via alanine substitution should not be able to enhance the stability of mentioned complex, we propose that R171 also serves other purposes. As exemplified in Fig. 6, the modeled distal C-terminal tail of arrestin-1 is able to orient itself in a way to enable interactions between R171 and negatively charged, C-terminal residues. Furthermore, the exposed positioning of R171 on top of the arrestin-1 N-domain qualifies this residue to serve as another anchor for the C-terminal tail, thus stabilizing the inactive arrestin-1 conformation. In a computational study performed by Sente *et al*. in^36^ various similarly multi-functional residues of arrestin-1 have been discussed^36^. To justify the complex stabilizing effect of the R171A mutation, the inhibiting effect of electrostatic interactions formed with R171 in the inactive arrestin-1 conformation has to surpass the complex stabilizing impact of interactions between R171 and phosphorylated GPCR residues. Our presented data and modeling efforts suggest that R171, which has been shown to bind to the leading phosphorylation of the GPCR C-terminal tail^26^, might also be important for rigidifying the inactive arrestin-1 conformation by association with negatively charged residues comprised in the arrestin-1 C-terminal tail.

## Experimental Procedures

### Combinatorial mutagenesis

Bovine arrestin-1 mutant F375A fused to fluorescent protein mCherry followed by 6xHis or Twin-Strep tag was selected for downstream engineering with PCR to generate more than 50 double mutants. Two double mutants were selected and further mutations sequentially introduced, yielding triple and quadruple mutants. The EgWoMiPi vector, PCR and program suite used for scanning mutagenesis are described elsewhere^14,42^.

### Preparation of rod outer segments in different states

Rod outer segment (ROS) disc membranes that are densely packed with rhodopsin were isolated from native source exactly as previously described^14,58^. A single-large scale ROS preparation was made, distributed in several aliquots and frozen at −80 °C. P-opsin and P-ROS were obtained following the rhodopsin phosphorylation procedure as previously described, taking advantage of endogenously available GRK1^13,14^, yielding a mixture of rhodopsin species containing any number of phosphates with up to seven phosphate groups per molecule whereas three phosphates are sufficient for high-affinity rhodopsin binding^59^.

### Binding assay

Mutants were expressed as described earlier^14^ and cells disrupted in buffer A [10 mM Hepes (pH 7.0), 100 mM NaCl, 1 mM DTT, 1 mM MgCl_2_, and 0.1 mM EDTA] or buffer B (containing 1.842 M NaCl), both supplemented with 0.2 mg/mL lysozyme, 20 μg/mL DNase, 1.5 mM PMSF, and protease inhibitor mixture Roche Complete. Further steps were carried out accordingly, as in^14^. In detail, 1.024 mL cleared cell lysate containing wild type or mutant arrestin-mCherry protein in buffer A was mixed with 76 μL ROS-P*, while 637.1 μL cleared cell lysate in buffer B was mixed with 82.9 μL of the same ROS-P* (1.45 mg/mL) stock, obtaining master mixes A or B, respectively. Master mix A was distributed in 100-μL portions to eight wells of a 96-well plate (in the following called plate A) with each well containing 100 μL buffer A with increasing amounts of sodium chloride, finally yielding 100, 247, 492, 737, and 982 mM and 1.472, 1.962, and 2.403 M NaCl in the eight reaction mixes. Each plate A contained wild type arrestin-mCherry for reference and eleven different arrestin-mCherry mutants. Master mix B was portioned in 60-μL fractions and transferred to eight wells of a 96-well plate (below called plate B) with each well containing 140 μL of the same buffer with different amounts of sodium chloride, resulting in 492, 737, and 982 mM and 1.472, 1.962, 2.403, 3.176, and 3.949 M NaCl. With each plate B arrestin-mCherry mutant F375A (as reference) and 11 different arrestin-mCherry mutants were assayed. Samples in each well were mixed, at 37 °C, incubated for 5 min and light activated for 6 min. Separate 96-well plates were filled with the following samples and processed in parallel in the dark: one 100-μL fraction of each master mix A was combined with 100 μL buffer A or one 60-μL portion of each master mix B with 140 μL buffer which was supplemented with NaCl to yield 492 mM NaCl. All plates were centrifuged and supernatants were removed as described^14^. The pellets were washed by carefully adding 100 μL buffer A to plates A or 100 μL buffer with 492 mM NaCl to plates B. Dark controls were treated accordingly. Pellets in plates A were resuspended with buffer A and pellets in plates B with buffer containing 492 mM NaCl. Quantification of pulled-down arrestin-mCherry was conducted as in^14^. SI Table 1 lists the constructed mutants and includes the number of measurements and thereof derived IC_50_ and R^2^ values as well as 95% confidence intervals.

### Arrestin purification from *E. coli*

Wild type and mutant arrestins fused to mCherry with C-terminal 6xHis-tag were expressed separately in *E. coli* strain BL21(DE3). Overnight cultures were diluted 1:50 in 12x 1 L LB medium (Gerbu Biotechnik) containing antibiotics. Expression was induced at an OD595 of 0.6–0.8 with 0.5 mM IPTG for about 18 hours at 20 °C. Harvested cell pellets were resuspended in 300 mL ice-cold lysis buffer composed of buffer C [50 mM Tris-HCl (pH 7.5), 500 mM NaCl, 10 mM imidazole, 5 mM β- mercaptoethanol] and 0.2 mg/mL lysozyme, 20 μg/mL DNase, and protease inhibitor mixture Roche Complete. Cells were cracked by pressure-assisted homogenization at 1,000 bar in four passages at 4 °C (EmulsiFlex-C3; Avestin). The suspension was centrifuged at 35,000 × g for 35 min at 4 °C (Optima XL-100K Ultracentrifuge, 45 Ti rotor; Beckmann Coulter) to remove cell debris. The supernatant was filtered using 0.45 μm filters (MF Membrane Filters; Millipore). The filtrate was loaded on a 5 mL Nickel affinity column (HisTrap FF crude; GE Healthcare) on a chromatography system (Äkta Express; GE Healthcare) with buffer C as the running buffer. The column was washed with buffer C containing 60 mM imidazole. His-tagged protein was eluted with buffer C containing 500 mM imidazole. The eluted fractions were analyzed by SDS-PAGE to identify arrestin-containing peaks. Selected fractions were pooled and concentrated with a 30-kDa cut-off concentrator (Amicon Ultra, Ultracel-30K; Millipore) to a volume of 4 mL. The sample was loaded onto a size-exclusion chromatography column (Superdex 200, 16/60; GE Healthcare) with running buffer [50 mM Hepes (pH 7.5), 150 mM NaCl, 5 mM β-mercaptoethanol]. Fractions were analyzed by SDS-PAGE and peaks corresponding to the size of arrestin-mCherry fusion protein pooled and concentrated as described above. Arrestin aliquots were flash-frozen in liquid nitrogen and stored at −80 °C.

### Arrestin-1 CPM thermal shift assay

Thermal induced unfolding of arrestin-1 using a thiol-sensitive dye was performed essentially the same as in^21^. 7- diethylamino-3-(4′-maleimidylphenyl)-4-methylcoumarin (CPM) (Life Technologies) is a weak fluorescent dye which becomes strongly fluorescent when covalently bound to exposed thiol groups^60^. We used arrestin in fusion with mCherry because mCherry does not contain any cysteins^61^. The CPM stock (3 mg/ml in DMSO) was diluted 1:40 into ice-cold buffer [10 mM Hepes (pH 7.0) and 100 mM NaCl]. In a cuvette, 5 μg purified arrestin-mCherry protein was profoundly mixed with 10 μL diluted CPM dye. The volume was adjusted to 120 μL with ice-cold buffer and the cuvette was placed into the fluorimeter (Cary Eclipse Fluorescence Spectrophotometer, Varian; Agilent Technologies). The sample was excited at 387 nm and emission was recorded at 464 nm. The temperature was ramped from 20 to 90 °C by 2 °C/min. Obtained data were fitted using a sigmoidal Boltzmann equation (Prism; GraphPad) and melting temperatures (T_M_) of arrestin-mCherry constructs were determined. The results from the four measurements were averaged.

### In-gel fluorescence thermo-stability assay

Arrestin-mCherry fusion proteins were expressed, harvested and lysed as described^14^. The cell lysate from a 50-mL cell-culture fraction was cleared by centrifugation (Centrifuge 5424 R; Eppendorf) at 21,100 × g for 20 min at 4 °C. The lysate was distributed in 100-μL portions to eleven 1.5-mL tubes (Sarstedt), which were put into a heating block that was equilibrated at 30 °C. The temperature was ramped up to 80 °C in 5 °C-steps manually at 2.5 min intervals. Samples were removed successively every 2.5 min and chilled on ice. Precipitant was removed by centrifugation for 1 h. 12 μL supernatant of each sample was mixed with 3 μL 5x SDS-loading dye. Full-length arrestin-mCherry construct was separated from degraded protein by SDS-PAGE for 1 h at 200 V and 80 mA in MOPS buffer using an 8–12% Bis-Tris gradient gel (Novex NuPAGE; Life Technologies) in supplied chamber (Novex NuPAGE SDS-PAGE gel system; Life Technologies). Fluorescence-emission of mCherry or mCherry-protein fusions was detected by exciting the protein at 312 or 365 nm using a 605 nm-filter (ImageQuant RT ECL; GE Healthcare). Fluorescence intensities were quantified by ImageJ (NIH) and plotted in Prism. Boltzmann sigmoidal fitting allowed to determine T_M_ and R^2^ values and standard errors.

### Expression of arrestin-1 in HEK293 cells

For every arrestin-1 mutant HEK293 cells were grown in 18 150 mm cell and tissue culture dishes (Biofil^®^) to ~60% confluence in DMEM High Glucose (4.5 g/l) medium with L-glutamine (Amimed^®^) containing 10% (v/v) FCS (FCS/FBS Sera Plus, seraglob.com) and a 1:100 dilution of penicillin/streptomycin (PS) (10000 IU/ml penicillin, 10000 µg/ml streptomycin; Amimed^®^) at 37 °C, 5% CO_2_. The cells were transiently transfected with 30 µg pcDNA3.1 harbouring the arrestin-1–mCherry-Strep-tag construct. The transfection mixture consisting of 30 µg DNA, 100 µl 1 mg/ml polyethylenimine (PEI) dissolved in water (25 kDa linear) (Sigma-Aldrich) and 12.5 ml DMEM high glucose (4.5 g/l) medium with L-glutamine was incubated for 10 min at room temperature. The growth medium was removed from the cells and 12.5 ml transfection mixture was added per dish. After incubation at 37 °C for 1 h, 5% CO_2_ 12.5 ml DMEM high glucose (4.5 g/l) medium with L-glutamine (10% (v/v) FCS, PS) was added. Expression was allowed for 2 days.

In order to harvest the cells, the medium was removed, cells were scraped from the bottom of the plate and suspended in cold PBS. The cell suspension was centrifuged for 5 min at 800 rpm and 4 °C. The supernatant was discarded, the cell pellet resuspended in cold PBS and centrifuged again for 5 min at 800 rpm and 4 °C. The supernatant was removed and the cell pellet was frozen at −80 °C.

### Arrestin purification from HEK293

Cell pellets were thawed and refrozen three times at −80 °C as to disrupt the cells. The cells were resuspended in washing buffer W150 (100 mM Tris (pH 7.5), 150 mM NaCl, 1 mM DTT, 1 mM EDTA, 1 mM PMSF) to a total volume of 5 ml. The cell suspension was sonicated on ice 10 × [10 s (amplitude 35, 0.5 s on/0.5 s off) + 10 s cooled down] and subsequently centrifuged for 30 min at 100k rcf (Beckman Coulter^®^ MLA-80 rotor in a Beckman Coulter^®^ Optima^TM^ MAX_XP ultracentrifuge) at 4 °C.

A Strep-Tactin^®^ Superflow^®^ high capacity 0.2 ml column (IBA) was washed with 4 ml washing buffer W150 before the supernatant from the previous centrifugation step was loaded. Subsequently, the column was washed with 3 ml washing buffer W150 and equilibrated with 1 mL buffer W100 (100 mM Tris (pH 7.5), 100 mM NaCl, 1 mM DTT, 1 mM EDTA, 1 mM PMSF). The protein was eluted in 950 µl elution buffer (100 mM Tris (pH 7.5), 100 mM NaCl, 1 mM DTT, 1 mM EDTA, 1 mM PMSF, 2.5 mM desthiobiotin) from which the first 150 µl were discarded.

An absorbance spectrum was measured in the range of 200 nm to 800 nm and the concentration was determined with the help of the following equation, where A is absorbance, ε the extinction coefficient of mCherry^61^, and d the path length of the light beam through the sample. A_587_ is the absorbance at ~587 nm:

$${\rm{c}}=\frac{{\rm{A}}}{{\rm{\varepsilon }}\times {\rm{d}}}=\frac{{{\rm{A}}}_{587}}{72000\,{{\rm{M}}}^{-1}{{\rm{cm}}}^{-1}\times 1\,{\rm{cm}}}\times 76079.58\,{\rm{g}}\,{{\rm{mol}}}^{-1}$$

The quality of the purified arrestin-1 was assessed by in-gel fluorescence on an SDS-gel. 1 µg arrestin-1 was loaded on a TruPAGE^TM^ Precast Gel 12% (10 × 10 cm, 12 well) (Sigma-Aldrich) and run in 1x Nu-PAGE^®^ SDS MES running buffer (Life Technologies) for 40 min at 180 V and 400 mA. mCherry in-gel fluorescence was measured on an Amersham Imager 600RGB. Using ImageJ, the intensity of the band of non-degraded arrestin-1 was set in relation to the intensity of the standard and the concentration previously determined on a spectrophotometer was corrected by multiplication with the resulting factor.

### Arrestin-1/(Rhod-)Opsin Pull-Down Assay

Each pull-down was carried out with 1.5 µg arrestin-1–mCherry fusion protein and 15 µg (rhod-)opsin contained in native membranes. Five different states of (rhod-)opsin were assessed: light-activated P-ROS (P-ROS*), P-ROS dark control, light-activated ROS (ROS*), ROS dark control, and P-opsin dark control. Arrestin-1 and ROS-membranes were mixed in the dark and equilibrated to a total volume of 50 µl using buffer D [100 mM Hepes pH 7.0, 100 mM NaCl, 1 mM DTT, 0.1 mM EDTA]. P-ROS* and ROS* samples were illuminated for 2.5 min (long-pass filter 495 nm). All samples were centrifuged at 15000 rpm at 4 °C for 30 min. The supernatant was transferred to a 96-well plate and the pellet was washed with 50 µl buffer D before a second centrifugation step at 15000 rpm at 4 °C for 30 min. The supernatant was again transferred to the 96-well plate as well as the pellet, which had been resuspended in 50 µl buffer D. Fluorescence intensity of mCherry was measured on a safire^2^ plate reader (Tecan) using the software Magellan^TM^ 6 (Tecan) (measurement parameters: excitation wavelength = 562 nm, emission wavelength = 612 nm, excitation and emission bandwidth = 20 nm, integration time = 40 µs, gain = 75).

### Modelling of the arrestin-1 C-terminal tail

A 3D model of bovine visual arrestin was built by homology modeling using the crystal structure of 1CF1^29^ as the structural template. The template was chosen for having resolved the longest C-terminal tail of all inactive visual arrestin crystal structures, while still lacking the most part of the distal C-terminus. The bovine arrestin-1 sequence (Uniprot:P08168) was used to build the model. MODELLER9.14 version^62,63^ was utilized in creating the model^62^. Initially, 100 models were assembled and all models were subjected to 300 iterations of variable target function method optimization and thorough molecular dynamics and simulated annealing optimization, and scored using the discrete optimized protein energy potential (DOPE).

Finally, each of the top ten scoring models were screened visually and the best one with optimal orientation of C-terminal was selected for the next step. Loop modelling was performed for the C-terminal part of the model (residues 355–404) and the one with best DOPE score was then again energy-minimized (5000 steps) and equilibrated using Implicit solvent (GBSW) modeler, CHARM-GUI and NAMD2.10^64,65^.

## Supplementary information

Supplementary information for arrestin-1 engineering facilitates complex stabilization with native rhodopsin
