# Supplementary material for: Arrestin-1 engineering facilitates complex stabilization with native rhodopsin
Source: Sci Rep. 2019 Jan 24;9:439. doi: 10.1038/s41598-018-36881-4 (PMC6346018; doi:10.1038/s41598-018-36881-4)
Supplement: Supplementary file 1 — Supplementary information for arrestin-1 engineering facilitates complex stabilization with native rhodopsin [file 41598_2018_36881_MOESM1_ESM.docx]

**Arrestin-1 engineering facilitates complex stabilization with native rhodopsin**

**Raphael S. Haider, Florian Wilhelm, Aurélien Rizk, Eshita Mutt, Xavier Deupi, Christian Peterhans, Jonas Mühle, Philipp Berger, Gebhard F.X. Schertler, Jörg Standfuss and Martin K. Ostermaier**

**MATERIALS INCLUDED**

SI-Table 1

| **Arrestin-1 mutants** | | | | | | |
| --- | --- | --- | --- | --- | --- | --- |
| **Mutant** | **IC_50_ [M]** | **95% confidence interval** | **Quality of fit (R^2^)** | **N° of curves determining IC_50_ value** | **Functional expression**  **level [%]** | **T_M_ (arrestin) [**°**C]  (N**° **of measurements)** |
| WT | 0.41 | 0.37 to 0.46 | 0.9937 | 74 | 100 ± 53 | 64.2 ± 0.6 (10) |
| V11A | 0.61 | 0.56 to 0.67 | 0.9982 | 2 | 50−150 | 61.6 ± 0.8 (2) |
| F13A | 0.84 | 0.74 to 0.95 | 0.9974 | 3 | 50−150 | 57.4 ± 1.7 (1) |
| Y25A | 0.59 | 0.51 to 0.71 | 0.9949 | 3 | 50−150 | 61.3 ± 0.4 (1) |
| V44A | 0.51 | 0.49 to 0.53 | 0.9994 | 2 | 50−150 | 64.1 ± 0.9 (1) |
| R66A | 0.64 | 0.59 to 0.69 | 0.9978 | 3 | 50−150 | 58.5 ± 0.7 (1) |
| Q87A | 0.54 | 0.50 to 0.59 | 0.9974 | 2 | 50−150 | weak signal |
| L111A | 0.70 | 0.64 to 0.76 | 0.9980 | 2 | 50−150 | 61.0 ± 0.6 (1) |
| C143A | 0.50 | 0.48 to 0.51 | 0.9997 | 2 | 50−150 | weak signal |
| G144A | 0.57 | 0.48 to 0.68 | 0.9921 | 2 | 50−150 | 59.8 ± 0.3 (1) |
| I149A | 0.74 | 0.64 to 0.85 | 0.9901 | 3 | ≤20 | weak signal |
| V170A | 0.58 | 0.51 to 0.66 | 0.9975 | 2 | 50−150 | 60.2 ± 0.8 (2) |
| R171A | 0.54 | 0.47 to 0.61 | 0.9962 | 2 | 50−150 | 60.2 ± 0.6 (2) |
| L173A | 0.51 | 0.47 to 0.55 | 0.9983 | 2 | 50−150 | 62.4 ± 1.0 (1) |
| R175A | 0.91 | 0.77 to 1.08 | 0.9948 | 3 | 50−150 | 52.5 ± 0.7 (2) |
| V247A | 0.59 | 0.55 to 0.62 | 0.9989 | 2 | 50−150 | 54.3 ± 1.2 (1) |
| R291A | 0.56 | 0.51 to 0.62 | 0.9967 | 2 | 50−150 | 63.1 ± 0.3 (1) |
| D296A | 0.92 | 0.71 to 1.21 | 0.9860 | 3 | 50−150 | 59.4 ± 1.1 (2) |
| G297A | 1.14 | 0.82 to 1.75 | 0.9879 | 3 | 50−150 | 52.6 ± 1.3 (1) |
| K298A | 0.52 | 0.41 to 0.66 | 0.9906 | 2 | 50−150 | 64.3 ± 0.3 (1) |
| D303A | 0.87 | 0.76 to 0.99 | 0.9964 | 3 | 50−150 | 55.2 ± 0.8 (1) |
| T304A | 0.84 | 0.74 to 0.96 | 0.9967 | 3 | 50−150 | 60.3 ± 1.3 (2) |
| A307G | 0.64 | 0.57 to 0.72 | 0.9967 | 2 | 50−150 | 57.3 ± 0.4 (3) |
| E341A | 0.57 | 0.52 to 0.63 | 0.9961 | 2 | 50−150 | 62.6 ± 1.2 (2) |
| F375A | 1.32 | 0.94 to 1.95 | 0.9849 | 23 | 107 ± 26 | 63.3 ± 1.0 (3) |
| V376A | 0.66 | 0.58 to 0.75 | 0.9957 | 3 | 50−150 | 62.4 ± 0.7 (1) |
| F377A | 1.08 | 0.82 to 1.43 | 0.9949 | 3 | ≥150 | 66.0 ± 1.3 (2) |
| F380A | 1.00 | 0.70 to 1.45 | 0.9903 | 3 | ≥150 | 55.2 ± 0.3 (1) |
| R382A | 0.97 | 0.76 to 1.24 | 0.9944 | 4 | 50−150 | 61.8 ± 0.9 (1) |
| H10A+F375A | 1.54 | 1.40 to 1.69 | 0.9967 | 2 | 50−150 |  |
| V11A+F375A | 0.99 | 0.64 to 1.67 | 0.9805 | 2 | 50−150 | 60.4 ± 0.7 (1) |
| F13A+F375A | 1.02 | 0.91 to 1.16 | 0.9968 | 2 | 50−150 |  |
| I24A+F375A | 1.39 | 0.95 to 2.02 | 0.9884 | 2 | ≤30 |  |
| Y25A+F375A | 1.10 | 0.92 to 1.31 | 0.9958 | 3 | 50−150 |  |
| D30A+F375A | 1.41 | 0.87 to 2.34 | 0.9893 | 3 | 50−150 |  |
| V44A+F375A | 1.69 | 1.07 to 2.80 | 0.9871 | 3 | ≤30 |  |
| P49A+F375A | 1.75 | 0.81 to 4.18 | 0.9660 | 3 | ≤30 |  |
| R56A+F375A | 1.45 | 1.14 to 1.85 | 0.9968 | 3 | 50−150 |  |
| V57A+F375A | 2.17 | 1.25 to 3.83 | 0.9743 | 3 | ≤30 |  |
| V59A+F375A | 1.92 | 1.29 to 2.96 | 0.9806 | 3 | ≤30 |  |
| A64G+F375A | 1.16 | 0.78 to 1.75 | 0.9907 | 3 | 50−150 |  |
| R66A+F375A | 2.04 | 1.08 to 3.89 | 0.9674 | 3 | 50−150 | 59.3 ± 0.8 (2) |
| R80A+F375A | - | - | - | 3 | <detection |  |
| F85A+F375A | 1.02 | 0.65 to 1.71 | 0.9883 | 2 | 50−150 |  |
| Q87A+F375A | 2.01 | 1.33 to 3.10 | 0.9890 | 2 | ≤30 |  |
| L107A+F375A | 1.27 | 0.81 to 2.00 | 0.9834 | 2 | ≤30 |  |
| L111A+F375A | 1.53 | 0.74 to 3.55 | 0.9796 | 2 | 50−150 |  |
| D124A+F375A | 1.08 | 0.87 to 1.37 | 0.9942 | 2 | 50−150 |  |
| C128A+F375A | 1.19 | 1.00 to 1.42 | 0.9975 | 2 | 50−150 |  |
| C143A+F375A | 1.03 | 1.00 to 1.18 | 0.9969 | 3 | 50−150 |  |
| G144A+F375A | 1.48 | 0.85 to 2.78 | 0.9888 | 3 | 50−150 |  |
| V145A+F375A | - | - | - | 3 | <detection |  |
| D146A+F375A | 1.53 | 1.30 to 1.80 | 0.9916 | 2 | 50−150 |  |
| F147A+F375A | - | - | - | 3 | <detection |  |
| I149A+F375A | 2.24 | 1.62 to 3.09 | 0.9844 | 2 | ≤30 | 49.5 ± 2.3 (2) |
| K163A+F375A | 1.19 | 0.65 to 2.16 | 0.9866 | 3 | 50−150 |  |
| V170A+F375A | 1.06 | 0.70 to 1.70 | 0.9866 | 4 | 50−150 | weak signal (1) |
| R171A+F375A | 1.73 | 1.29 to 2.34 | 0.9844 | 2 | 50−150 |  |
| L173A+F375A | 1.58 | 1.34 to 1.87 | 0.9921 | 2 | 50−150 |  |
| R175A+F375A | 2.48 | 1.55 to 4.21 | 0.9723 | 3 | 50−150 |  |
| D183A+F375A | 0.98 | 0.67 to 1.43 | 0.9927 | 1 | 50−150 |  |
| V222A+F375A | 0.44 | 0.39 to 0.49 | 0.9949 | 1 | ≤30 |  |
| A225G+F375A | 1.42 | 0.73 to 3.51 | 0.9738 | 2 | 50−150 |  |
| V247A+F375A | - | - | - | 3 | ≤30 |  |
| D253A+F375A | 1.22 | 1.00 to 1.50 | 0.9968 | 3 | 50−150 |  |
| I256A+F375A | 1.23 | 0.83 to 1.84 | 0.9866 | 2 | 50−150 |  |
| K257A+F375A | 1.45 | 1.11 to 1.91 | 0.9947 | 3 | 50−150 |  |
| A261G+F375A | 1.46 | 0.84 to 2.74 | 0.9897 | 3 | 50−150 |  |
| S272A+F375A | 1.57 | 1.35 to 1.84 | 0.9931 | 2 | 50−150 |  |
| R291A+F375A | 1.42 | 0.91 to 2.40 | 0.9850 | 3 | 50−150 | 62.5 ± 0.6 (2) |
| D296A+F375A | 2.51 | 1.39 to 4.62 | 0.9604 | 2 | ≤30 | 56.1 ± 1.8 (1) |
| G297A+F375A | 1.34 | 0.84 to 2.20 | 0.9420 | 2 | ≤30 |  |
| K298A+F375A | 1.60 | 0.87 to 3.07 | 0.9829 | 4 | 50−150 | 61.1 ± 0.3 (2) |
| D303A+F375A | 1.46 | 0.85 to 2.89 | 0.9841 | 4 | 50−150 | 62.4 ± 0.7 (1) |
| T304A+F375A | 1.51 | 0.69 to 3.34 | 0.9848 | 2 | ≤30 | 58.2 ± 2.8 (2) |
| A307G+F375A | 2.83 | 1.38 to 5.78 | 0.9569 | 1 | 50−150 | 50.8 ± 4.2 (2) |
| E341A+F375A | 2.06 | 1.53 to 2.80 | 0.9904 | 3 | 50−150 | 58.1 ± 1.3 (3) |
| H357A+F375A | 0.86 | 0.50 to 1.47 | 0.9855 | 2 | 50−150 |  |
| N374A+F375A | 1.09 | 0.87 to 1.36 | 0.9968 | 2 | 50−150 | 57.8 ± 0.6 (2) |
| V376A+F375A | 1.21 | 0.93 to 1.59 | 0.9899 | 3 | 50−150 |  |
| F380A+F375A | 1.39 | 0.97 to 2.11 | 0.9874 | 2 | 50−150 | 57.3 ± 0.7 (2) |
| R382A+F375A | 1.44 | 0.93 to 2.27 | 0.9957 | 2 | 50−150 | 61.3 ± 1.1 (1) |
| H10A+T304A+F375A | 1.58 | 1.13 to 2.38 | 0.9895 | 2 | 50−150 |  |
| V11A+T304A+F375A | 1.29 | 0.97 to 1.74 | 0.9888 | 3 | 50−150 |  |
| D30A+T304A+F375A | 1.53 | 1.12 to 2.11 | 0.9845 | 2 | 50−150 |  |
| R80A+T304A+F375A | 1.50 | 0.81 to 2.75 | 0.9363 | 2 | 50−150 |  |
| D82A+T304A+F375A | 1.32 | 0.85 to 2.21 | 0.9811 | 3 | 50−150 |  |
| V90A+T304A+F375A | 2.07 | 1.60 to 2.68 | 0.9691 | 2 | 50−150 |  |
| L111A+T304A+F375A | 1.96 | 1.33 to 2.96 | 0.9770 | 2 | 50−150 |  |
| P123A+T304A+F375A | 2.02 | 1.46 to 2.79 | 0.9887 | 2 | 50−150 |  |
| C143A+T304A+F375A | 1.39 | 1.16 to 1.67 | 0.9956 | 2 | 50−150 |  |
| G144A+T304A+F375A | 1.93 | 1.09 to 4.68 | 0.9837 | 2 | 50−150 |  |
| D146A+T304A+F375A | 1.94 | 1.57 to 2.40 | 0.9907 | 3 | 50−150 |  |
| I149A+T304A+F375A | - | - | - | 3 | <detection |  |
| V170A+T304A+F375A | 1.22 | 1.00 to 1.48 | 0.9988 | 2 | 50−150 |  |
| L173A+T304A+F375A | 1.53 | 1.07 to 2.22 | 0.9969 | 2 | 50−150 |  |
| R175A+T304A+F375A | 1.51 | 1.13 to 2.02 | 0.9784 | 1 | ≤30 |  |
| V247+T304A+F375A | 1.01 | 0.57 to 1.89 | 0.9936 | 2 | ≤30 |  |
| D296A+T304A+F375A | 1.39 | 1.06 to 1.85 | 0.9941 | 2 | 50−150 |  |
| G297A+T304A+F375A | 1.25 | 1.02 to 1.53 | 0.9975 | 1 | 50−150 |  |
| K298A+T304A+F375A | 2.01 | 1.42 to 2.85 | 0.9832 | 3 | 50−150 |  |
| D303A+T304A+F375A | 2.45 | 1.50 to 4.26 | 0.9698 | 2 | 50−150 |  |
| A307G+T304A+F375A | 2.89 | 1.10 to 9.37 | 0.9446 | 2 | ≤30 |  |
| E341A+T304A+F375A | 2.75 | 1.19 to 6.37 | 0.9481 | 1 | 50−150 | 60.8 ± 1.5 (2) |
| V376A+T304A+F375A | 2.14 | 1.25 to 4.39 | 0.9822 | 2 | 50−150 |  |
| F380A+T304A+F375A | 2.10 | 1.00 to 5.95 | 0.9664 | 2 | 50−150 | 58.9 ± 0.6 (4) |
| R382A+T304A+F375A | 2.71 | 1.64 to 4.74 | 0.9493 | 2 | ≤30 |  |
| V11A+A307G+F375A | 2.40 | 1.37 to 4.23 | 0.9591 | 1 | 50−150 |  |
| R66A+A307G+F375A | 3.37 | 1.20 to 9.48 | 0.9544 | 1 | ≤30 |  |
| L111A+A307G+F375A | 2.16 | 0.76 to 9.30 | 0.9842 | 2 | ≤30 |  |
| P123A+A307G+F375A | 2.67 | 1.65 to 4.32 | 0.9700 | 1 | ≤30 |  |
| I149A+A307G+F375A | - | - | - | 1 | <detection |  |
| R175A+A307G+F375A | - | - | - | 1 | <detection |  |
| V247A+A307G+F375A | 3.41 | 1.48 to 7.88 | 0.9421 | 1 | ≤30 |  |
| R291A+A307G+F375A | 2.69 | 2.01 to 3.61 | 0.9883 | 1 | ≤30 |  |
| D296A+A307G+F375A | 2.42 | 1.96 to 2.99 | 0.8012 | 1 | ≤30 |  |
| G297A+A307G+F375A | 3.52 | 0.71 to 17.37 | 0.9228 | 1 | ≤30 |  |
| V376A+A307G+F375A | 2.33 | 1.25 to 4.51 | 0.9836 | 2 | 50−150 |  |
| F380A+A307G+F375A | 1.98 | 1.04 to 3.88 | 0.9062 | 2 | 50−150 |  |
| R382A+A307G+F375A | 3.11 | 1.29 to 7.52 | 0.9290 | 1 | 50−150 |  |
| V11A+E341A+T304A+F375A | 2.10 | 0.87 to 5.62 | 0.9660 | 3 | 50−150 | 60.3 ± 0.5 (1) |
| R66A+E341A+T304A+F375A | 2.59 | 1.07 to 6.74 | 0.9507 | 2 | 50−150 | 55.6 ± 0.6 (1) |
| R171A+E341A+T304A+F375A | 2.95 | 1.23 to 7.85 | 0.9405 | 2 | 50−150 | 58.9 ± 0.4 (1) |
| R291A+E341A+T304A+F375A | 2.10 | 1.05 to 4.31 | 0.9591 | 3 | 50−150 | 60.8 ± 1.3 (1) |
| K298A+E341A+T304A+F375A | 2.24 | 1.14 to 4.47 | 0.9741 | 3 | 50−150 | 61.3 ± 0.6 (1) |
| D303A+E341A+T304A+F375A | 2.91 | 0.85 to 9.99 | 0.9105 | 1 | 50−150 | 59.0 ± 0.7 (1) |
| R382A+E341A+T304A+F375A | 2.48 | 1.26 to 4.92 | 0.9714 | 3 | 50−150 | 55.7 ± 0.5 (1) |
| V11A+F380A+T304A+F375A | 1.63 | 1.14 to 2.33 | 0.9852 | 3 | 50−150 |  |
| R66A+F380A+T304A+F375A | 2.64 | 0.80 to 8.84 | 0.9603 | 2 | 50−150 | 58.0 ± 0.2 (1) |
| F85A+F380A+T304A+F375A | 1.78 | 0.96 to 3.62 | 0.9726 | 3 | 50−150 | 60.9 ± 0.6 (1) |
| R171A+F380A+T304A+F375A | 2.07 | 0.75 to 6.59 | 0.9408 | 3 | 50−150 | 55.9 ± 1.1 (1) |
| R291A+F380A+T304A+F375A | 1.92 | 0.90 to 4.35 | 0.9714 | 2 | 50−150 | 60.6 ± 0.4 (1) |
| K298A+F380A+T304A+F375A | 1.37 | 1.02 to 1.87 | 0.9868 | 2 | 50−150 | 61.1 ± 0.5 (1) |
| D303+F380A+T304A+F375A | 1.94 | 0.94 to 4.03 | 0.9678 | 2 | 50−150 | 60.2 ± 1.1 (1) |
| E341A+F380A+T304A+F375A | 2.00 | 0.91 to 5.39 | 0.9598 | 2 | 50−150 | 57.4 ± 0.3 (1) |

**SI Table 1.** List of constructed mutants that were screened for half-maximal inhibitory concentration (IC_50_) of NaCl to disrupt formation of complexes with P-ROS*. Binding of each mutant to P-ROS* in its natural environment, the rod outer segment (ROS) membranes, was quantified in eight different sodium chloride concentrations, ranging from 100 to 2403 mM. The measurement was repeated for the range from 492 to 3949 mM salt if the fitted sigmoidal dose-response curve could not reach the bottom plateau. The number of test sets is indicated from which IC_50_, R^2^ and 95% confidence interval were derived. It is remarked if expression of functional arrestin protein was too low to determine IC_50_ values reliably. The melting temperature (T_M_) of arrestin mutants was determined by an in-gel fluorescence assay.

SI-Figure 1

**
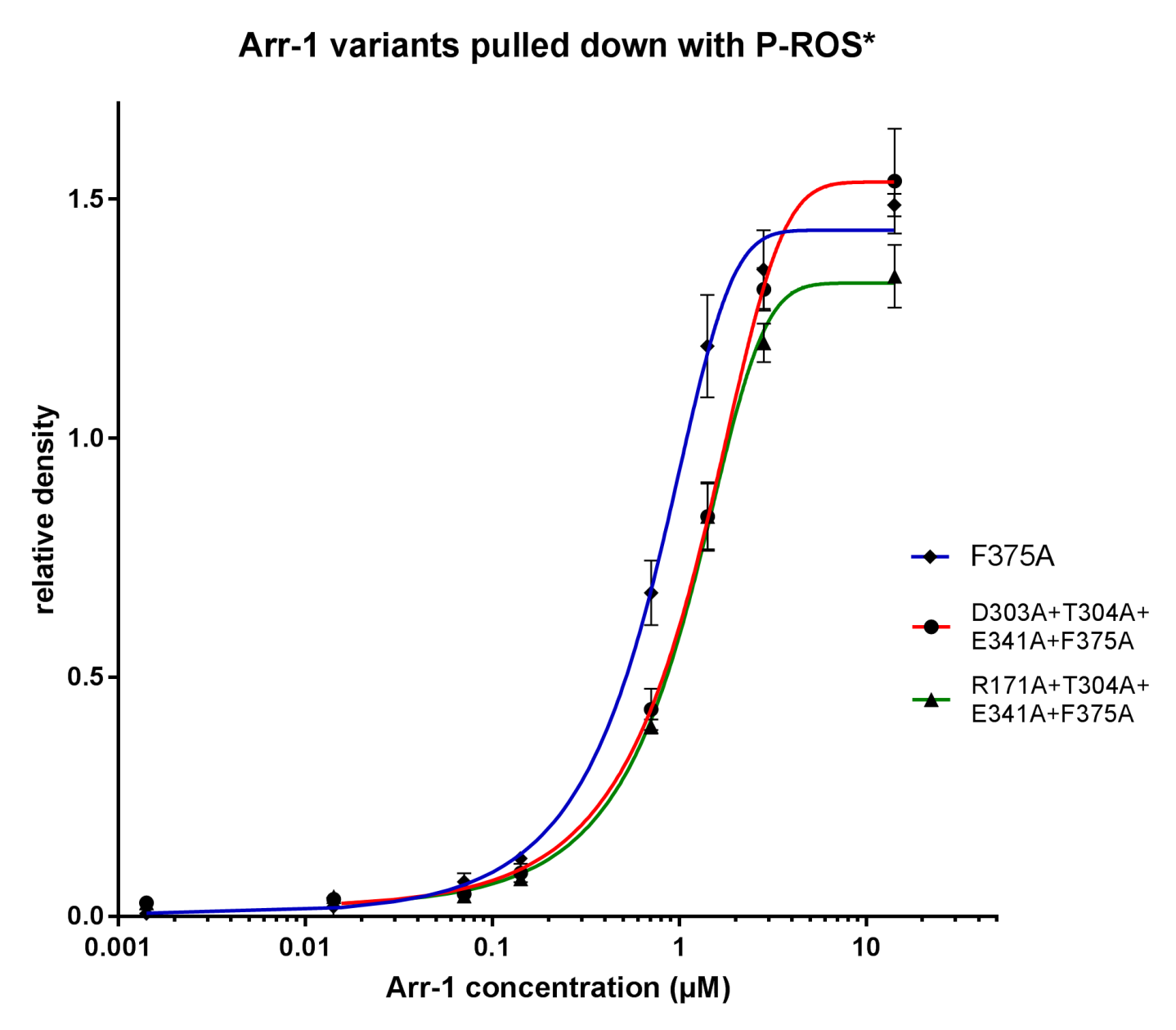
SI Figure 1.** F375A, D303A+E341A+T304A+F375A and R171A+E341A+T304A+F375A arrestin-1 mutant proteins were expressed in *E. coli* and purified. Each protein was used in various concentrations in a direct binding assay with membranes containing P-ROS* at a final concentration of 1.71 µM. The depicted relative density reflects the amount of arrestin-1 protein after membrane pull down and two washing steps separated by SDS-PAGE and visualized by subsequent Coomassie staining. The pull-down was carried out as described in the materials and methods section, in detail the following reaction conditions were used: 10 mM HEPES, 300 mM NaCl, pH 7.0. Each pull-down reaction was adjusted to a final volume of 150 µL. The experiments were carried out in triplicates and normalized to the amount of rhodopsin visualized with each individual pull-down. The error bars indicate standard deviations.
